# Democratizing calcium visualization

**DOI:** 10.1016/j.jbc.2021.101181

**Published:** 2021-09-08

**Authors:** Laurel Oldach

At a time when cloning a gene was a feat worthy of a high-impact publication, someone asked Roger Tsien why he studied calcium.

“His somewhat flippant answer was, ‘Because it cannot be cloned,’” recalled Joseph Kao, who was a postdoc in Tsien’s lab. “Early on, he was actually somewhat dismissive of molecular biology.”

Ironically, Tsien, who died in 2016, is best remembered for his contributions to developing a molecular biology icon: green fluorescent protein. His group’s work helped transform the protein from a coelenterate curiosity to a laboratory staple and earned Tsien a third of the 2008 Nobel Prize in Chemistry. Before launching that project in the 1990s, Tsien had already revolutionized the field of calcium sensing.

In the 1970s, Kao said, measuring calcium was “a very rarefied, arcane art” that depended on a deep knowledge of electrophysiology. Today, researchers can use a variety of fluorescent indicators to visualize the activity of calcium and other second messengers in living cells. Many modern indicators derive from a series of probes that Tsien’s lab developed.

In one JBC Classic article on such probes, “Ca^2+^ indicators based on fluoresceins and rhodamines,” Akwasi Minta, Kao, and Tsien introduced several fluorescent indicators of Ca^2+^ concentration that could be used in cells (1).

Tsien began to pursue an interest in calcium signaling early in his scientific career. The ion was already known as an important signal carrier central to muscle contraction, synaptic transmission, and many other physiological functions (2).

“Calcium was central to everything—but it was very difficult to measure,” Kao said. Tsien considered electrophysiology as an undergraduate, but by the time he started his graduate research at Cambridge, he was focused on chemical approaches.

The first Ca^2+^ chelator Tsien developed, 2-bis(*o*-aminophenoxy)ethane-*N*,*N*,*N′*,*N′*-tetraacetic acid, or BAPTA (3), remains widely used because of its rapid binding kinetics, high selectivity for calcium, and insensitivity to pH changes in the physiological range. BAPTA binds calcium through four carboxylate groups (Fig. 1). As a professor at the University of California, Berkeley, Tsien led a lab that developed the calcium probes fura-2 and indo-1 (4, 5), which are elaborations of the BAPTA architecture. Both of these molecules are intrinsically fluorescent but, upon binding Ca^2+^, the shapes of their fluorescence spectra change. The ratio of the fluorescence intensities at two different wavelengths can be calibrated into actual Ca^2+^ concentration. Because such ratiometric measurements are insensitive to the concentration of the indicator in the sample, some common experimental artifacts are minimized. Still, Tsien was disappointed that they required long-wavelength UV excitation, which can potentially harm cells and excite autofluorescence. Being a perfectionist, Kao said, Tsien was initially reluctant to publish on the new indicators but ultimately yielded to persuasion by postdoc Martin Poenie.

**Figure 1:**
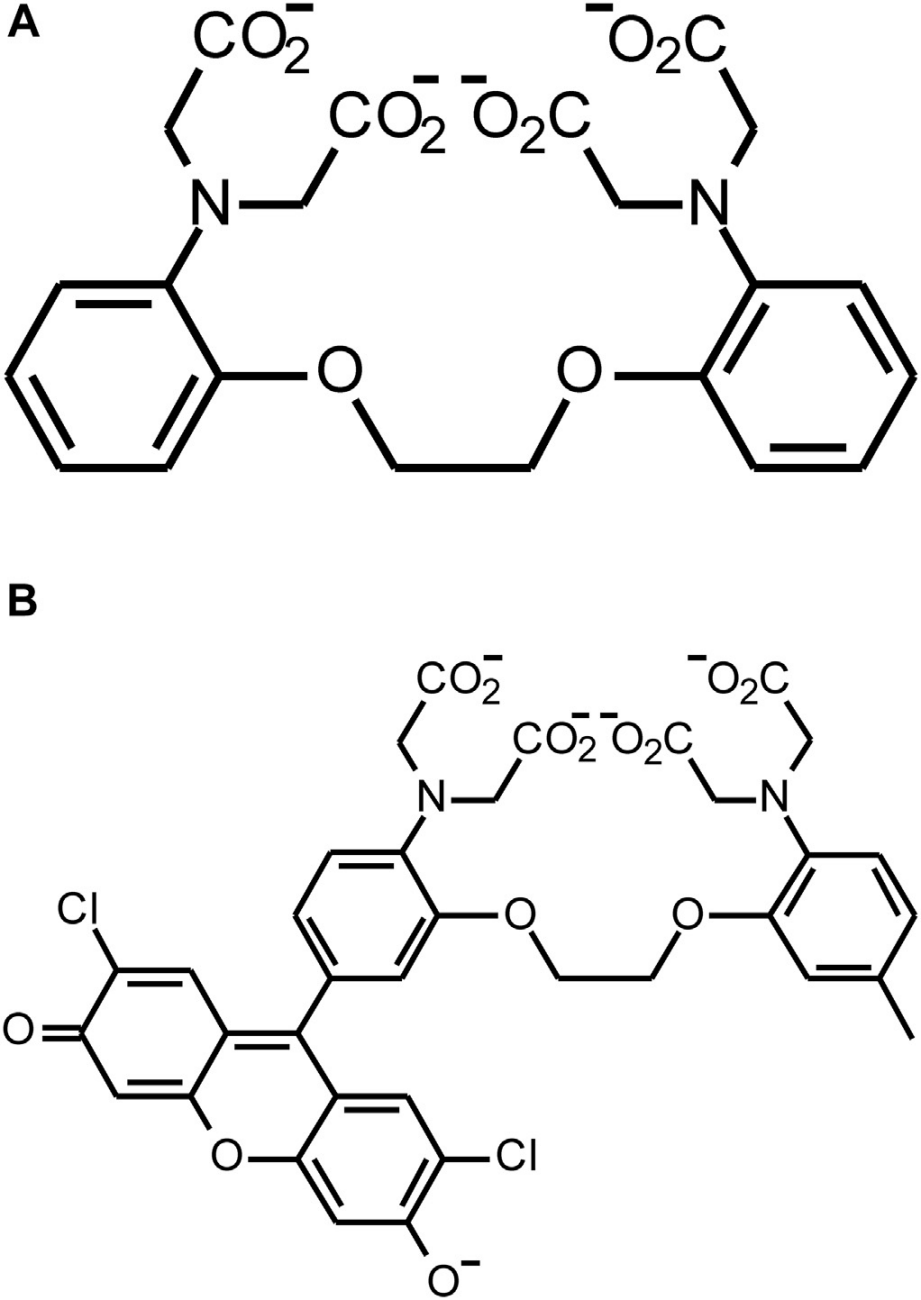
**Structures of BAPTA and Fluo3.** When unprotonated, the carboxylic acid groups in both BAPTA (*A*) and Fluo3 (*B*) can coordinate Ca^2+^.

Tsien was intent on developing a fluorescent Ca^2+^ indicator with excitation in the visible range. Perhaps, it could be done by combining BAPTA with the visible fluorescence of already-available fluorophores. He recruited postdocs Minta, a chemist, and Kao, a biophysicist, to work on the project.

First, the molecules had to be synthesized. An early synthetic strategy involved a long pressurized incubation in an aluminum instrument that resembled an old-fashioned coffee urn. Kao recalled a time when the chemists left a reaction to run over the weekend, hoping to improve its yield.

“When we came back… there was an imprint where the lid handle had smashed into the ceiling. Somewhere else, we found the lid completely flattened.” The reaction also had shattered an internal glass ampule, leaving glass dust all over the lab. Kao added, “Subsequently, they found better ways to do that reaction.”

Even after the molecules were synthesized, the work was not complete. “Roger was a perfectionist,” Minta said. “If I did a dye and it had certain imperfections, he made me start all over again.”

Some molecules he generated failed to fluoresce or were protonated at a near-physiological pH. Minta tweaked and adapted, adding and modifying functional groups until he had two chimeric molecules, derived from the fluorophores rhodamine and fluorescein, which were weakly fluorescent on their own but lit up dramatically when Ca^2+^ bound (Fig. 1). The lab dubbed the probes fluo-3 and rhod-2.

Ordinarily when a molecule absorbs light, the energy is quickly dispersed as molecular motion, or heat. Fluorescence—the release of captured light energy as a photon—requires special circumstances.

“When a molecule absorbs light, an electron is promoted from a lower-energy level into a higher-energy level, leaving a vacancy in the lower level,” Kao said. Emission of a photon depends on the excited electron returning to its normal, lower-energy state. If an electron elsewhere in the molecule is free to slip into that lower orbital, he said, the excited electron’s energy is ultimately lost as heat instead of being emitted as light, a phenomenon known as fluorescence quenching.

In the chimeric molecules, the fluorophore can be quenched by electrons in lone pairs on the BAPTA moiety (Fig. 2). But when a positively charged Ca^2+^ is present, it forms bonds with those electrons, lowering their energy and making it energetically unfavorable for them to fill the vacancy left by the excited electron. Without competition for the vacated orbital, the excited electron can relax back into it, emitting a photon.

**Figure 2:**
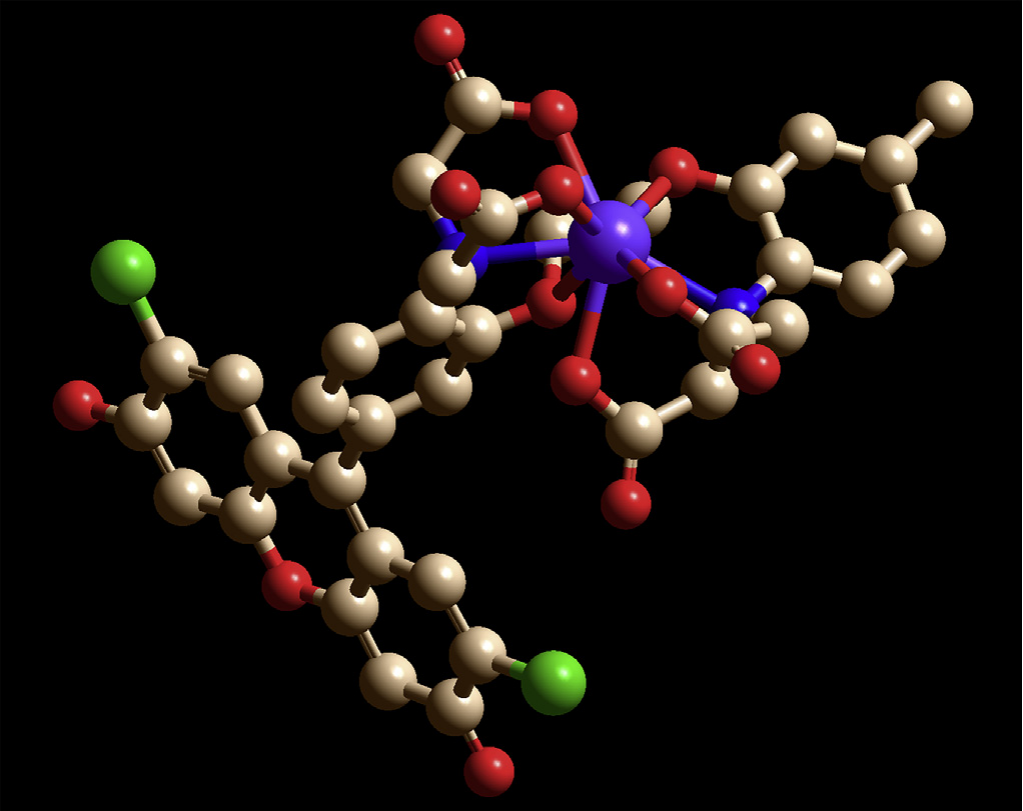
**Three-dimensional model of Fluo3 bindingCa**^**2+**^**(*purple*).** The structures show the participation of the two nitrogens (*blue*) and six oxygens (*red*) that bind to Ca^2+^, causing Ca^2+^ to be enveloped by the chelator.

“Calcium allows it to fluoresce beautifully,” Minta said. In a test tube, fluo-3’s brightness increased by 40- to 100-fold when calcium was added.

In a second article in the same issue of JBC, Kao and several colleagues tested the probes for live-cell imaging (6). They found that cells took up the indicators through incubation with the corresponding acetoxymethyl (AM) esters and confirmed a dazzling increase in fluorescence when they applied Ca^2+^-mobilizing agonists.

Fluo-3 was quickly adopted for many uses. By 1995, researchers had reported watching waves of Ca^2+^ activity pass through connected networks of neurons in mouse brain slices, observing cell-cycle initiation in fertilized egg cells and detecting “Ca^2+^ sparks”—microscopic, elementary Ca^2+^ signals generated by the coordinated opening of small clusters of Ca^2+^-release channels on the sarcoplasmic reticulum in heart cells (7, 8, 9). Kao said that the new technologies “made calcium measurement accessible to essentially anyone with a microscope.”

Still, Tsien was disappointed that the new probes changed only in intensity, not in excitation or emission wavelength, upon Ca^2+^ binding; he had hoped to be able to do ratiometric imaging.

“Roger was almost always dissatisfied with any product that you made,” Kao said. “He had a perfect conception of how they should behave, and then they would fall short on one or another aspect, and he’d be a little rueful: ‘If only we had discovered how to do this.’”

Tsien did finally get his visible ratiometric Ca^2+^ sensor about 10 years later. It was based on GFP and the calcium-binding protein calmodulin; although calcium could not be cloned, cloning turned out to be useful in its study after all (10).

## References

[1] Minta A., Kao J.P., Tsien R.Y. (1989). Fluorescent indicators for cytosolic calcium based on rhodamine and fluorescein chromophores. J. Biol. Chem..

[2] Tsien R.W., Tsien R.Y. (1990). Calcium channels, stores, and oscillations. Annu. Rev. Cell Biol..

[3] Tsien R.Y. (1980). New calcium indicators and buffers with high selectivity against magnesium and protons: design, synthesis, and properties of prototype structures. Biochemistry.

[4] Grynkiewicz G., Poenie M., Tsien R.Y. (1985). A new generation of Ca2+ indicators with greatly improved fluorescence properties. J. Biol. Chem..

[5] Kresge N., Simoni R.D., Hill R.L. (2006). The chemistry of fluorescent indicators: the work of Roger Y. Tsien. J. Biol. Chem..

[6] Kao J.P., Harootunian A.T., Tsien R.Y. (1989). Photochemically generated cytosolic calcium pulses and their detection by fluo-3. J. Biol. Chem..

[7] Dani J.W., Chernjavsky A., Smith S.J. (1992). Neuronal activity triggers calcium waves in hippocampal astrocyte networks. Neuron.

[8] Kline D., Kline J.T. (1992). Repetitive calcium transients and the role of calcium in exocytosis and cell cycle activation in the mouse egg. Dev. Biol..

[9] Cheng H., Lederer W.J., Cannell M.B. (1993). Calcium sparks: elementary events underlying excitation-contraction coupling in heart muscle. Science.

[10] Miyawaki A., Llopis J., Heim R., McCaffery J.M., Adams J.A., Ikura M., Tsien R.Y. (1997). Fluorescent indicators for Ca2+ based on green fluorescent proteins and calmodulin. Nature.

